# Enhancing surgical outcomes in elderly gastric cancer patients: the role of comprehensive preoperative assessment and support

**DOI:** 10.1186/s12957-024-03421-6

**Published:** 2024-05-23

**Authors:** Yuki Ushimaru, Shinnosuke Nagano, Ryohei Kawabata, Kazuhiro Nishikawa, Tomohira Takeoka, Akihiro Kitagawa, Nobuyoshi Ohara, Hideo Tomihara, Sakae Maeda, Mitsunobu Imasato, Shingo Noura, Atsushi Miyamoto

**Affiliations:** 1 Department of Gastroenterological Surgery, Sakai City Medical Center, 1-1-1 Ebaraji-Cho, Nishi-Ku, Sakai City, Osaka, 593-8304 Japan; 2 Department of Gastroenterological Surgery, Osaka International Cancer Institute, Osaka, Japan; 3 Department of Next Generation Endoscopic Intervention (Project ENGINE), Osaka University Graduate School of Medicine, Osaka, Japan

**Keywords:** Gastric cancer, Elderly patients, Geriatric assessment, Preoperative support

## Abstract

**Background:**

As the prevalence of gastric cancer rises in aging populations, managing surgical risks and comorbidities in elderly patients presents a unique challenge. The Comprehensive Preoperative Assessment and Support (CPAS) program, through comprehensive preoperative assessments, aims to mitigate surgical stress and improve outcomes by enhancing patient awareness and preparation. This study investigates the efficacy of a CPAS program, incorporating frailty and sarcopenia evaluations, to improve short-term outcomes in elderly gastric cancer patients.

**Methods:**

A retrospective analysis was conducted on 127 patients aged 75 or older who underwent surgery with CPAS between 2018 and August 2023, compared to 170 historical controls from 2012 to 2017. Propensity score matching balanced both groups based on age-adjusted Charlson Comorbidity Index and surgical details. The primary focus was on the impact of CPAS elements such as rehabilitation, nutrition, psychological support, oral frailty, and social support on short-term surgical outcomes.

**Results:**

Among 83 matched pairs, the CPAS group, despite 40.4% of patients in the CPAS group and 21.2% in the control group had an ASA-PS score of 3 or higher (*P* < 0.001), demonstrated significantly reduced blood loss (100 ml vs. 190 ml, *P* = 0.026) and lower incidence of serious complications (19.3% vs. 33.7%, *P* = 0.034), especially in infections and respiratory issues. Sarcopenia was identified in 38.6% of CPAS patients who received tailored support. Additionally, the median postoperative hospital stay was notably shorter in the CPAS group (10 days vs. 15 days, *P* < 0.001), with no in-hospital deaths. These results suggest that personalized preoperative care effectively mitigates operative stress and postoperative complications.

**Conclusion:**

Implementing CPAS significantly enhances surgical safety and reduces complication rates in elderly gastric cancer patients, emphasizing the critical role of personalized preoperative care in surgical oncology for this demographic.

## Background

The global rise in aging populations has led to an increased incidence of gastric cancer among the elderly [1–3], presenting distinct challenges for healthcare providers. While surgery remains the primary treatment, the advanced age of these patients often corresponds with increased surgical risks and comorbidities, necessitating a nuanced treatment approach. It is critical to balance the potential benefits of surgery against the risks of postoperative complications in this demographic [4–8]. Effective management of these complications is essential not only to improve immediate surgical outcomes but also to reduce non-cancer mortality, further underscoring the importance of postoperative care [9]. The management of these patients requires a nuanced understanding of their unique health profiles to optimize surgical decisions and improve prognoses. Notably, randomized controlled trials have highlighted the benefits of geriatric interventions. For instance, previous study demonstrated that perioperative geriatric care significantly reduces hospital stays and ICU usage in these patients [10], underscoring the value of comprehensive preoperative assessments. This approach is crucial in addressing the diverse medical needs of this population and ensuring favorable surgical outcomes [4, 11].

Technological advancements, especially minimally invasive techniques like laparoscopic and robotic surgery, offer potential benefits for the elderly by reducing operative stress and complications. However, these methods may not be suitable for all elderly patients [4–7]. The variability in health status, life expectancy, and patient preferences further complicates decision-making, requiring a personalized approach to care [12–14]. The diversity within the elderly population indicates that a one-size-fits-all approach is inadequate. A multidisciplinary team is essential to thoroughly assess and prepare each patient for surgery. This team should consider the patient's overall health, nutritional status, and comorbid conditions to optimize outcomes and minimize risks. Such a comprehensive evaluation is crucial to making informed decisions about whether to proceed with surgery and, if so, what type of procedure to undertake [4–8].

Despite the inherent risks, with careful patient selection and tailored surgical planning, elderly patients can achieve favorable outcomes following gastric cancer surgery. Continued research and the development of age-specific guidelines are imperative to enhance the care and prognosis of this growing patient population. As medical professionals, understanding and addressing the unique needs of elderly gastric cancer patients is paramount in improving their quality of life and survival rates [4–7]. The effectiveness of comprehensive preoperative assessment and support (CPAS), including evaluations for frailty and sarcopenia, in elderly gastric cancer patients for improving short-term outcomes has been uncertain. Recent studies have shown significant reductions in postoperative hospital stays and ICU utilization when geriatric assessments are integrated into perioperative care [10]. These challenges include increased sensitivity to treatment, slower recovery rates, and a higher incidence of comorbidities, necessitating a nuanced approach to treatment.

This study aims to investigate the effects of preoperative comprehensive assessments and supports on the short-term outcomes of elderly gastric cancer patients. By examining the impact of these preoperative strategies, we seek to provide evidence for their efficacy in improving surgical outcomes in this vulnerable patient population.

## Patients and methods

### Patients

Patients in this study were informed about the research's nature and purpose and provided informed consent. Conducted at Sakai City Medical Center, this study utilized a prospective database to extract information. The study was approved by the Ethics Committee of Sakai City Medical Center (approved number: 23–401). Consent for participation in the study was obtained using an opt-out approach.

Patients eligible for the CPAS program were prospectively enrolled starting in January 2018. The program included a questionnaire-based screening and evaluations for sarcopenia, followed by multidisciplinary interventions tailored to each patient's needs. A flowchart detailing these steps and the support provided has been added to this section for clarity. From January 2012 to August 2023, 584 patients diagnosed with gastric cancer and treated with surgical methods like distal gastrectomy (DG), proximal gastrectomy (PG), and total gastrectomy (TG) were included in this study. Cases from January 2018 onwards underwent comprehensive assessment, medical support, and patient education, designated as the CPAS group. As a historical control, cases from January 2012 to December 2017 were considered and referred to as the Historical control group. Surgical approaches included both open surgery (OS) and minimally invasive surgery (MIS), which encompassed laparoscopic and robotic surgery methods. This study specifically focused on elderly patients aged 75 years and above. All cases were histologically confirmed as gastric cancer. The TNM classification followed the guidelines set by the Japanese Gastric Cancer Association [15, 16]. All treatments were administered in accordance with the Japanese Gastric Cancer Treatment Guidelines [17, 18]. The decline in patient numbers in the more recent cohort can be attributed to a nationwide decrease in gastric cancer incidence due to Helicobacter pylori eradication and a significant reduction in diagnostic rates during the 2020 global COVID-19 pandemic [19–21].

Data for the selected cases were collated from the aforementioned prospective database for analysis. To adjust for background factors between the CPAS and Historical control groups, Propensity score matching analysis was conducted. Covariates used included Age-adjusted Charlson Comorbidity Index (aCCI) [17–20], sex, Body mass index (BMI), clinical T status (T1/2–4), clinical N status (-/ +), clinical M status, type of resection (TG/non-TG), approach (OS/MIS), and modified Glasgow Prognostic Score (0,1,2). The perioperative outcomes were examined for both groups of matched pairs.

### Comprehensive Preoperative Assessment and Support (CPAS) services

Sarcopenia was evaluated using the AWGS 2019 criteria [22]. Skeletal muscle mass was measured using bioelectrical impedance analysis (BIA) with a body composition analyzer (InBody, Seoul, Korea), and physical function was assessed by walking speed. As part of our study's methodology, the CPAS program began with comprehensive geriatric assessments (CGA) to evaluate each patient’s medical, psychological, and functional capabilities [23, 24]. This informed the integration of several tailored support services, structured around multidisciplinary collaboration and aimed at optimizing patient readiness and recovery (Fig. 2). These services were provided both on an outpatient basis and during the perioperative period. The details of these services are as follows:

1. *Rehabilitation Services*: Initiated during the outpatient visits, rehabilitation was led by physical therapists who conducted comprehensive evaluations to identify areas of physical weakness. Customized exercise programs were designed for each patient, which could be performed at home, with adjustments made based on the patient's recovery stage during follow-up visits. Post-operatively, the focus was on enhancing mobility and reducing recovery time, guided by initial assessments.
2. *Nutritional Support:* Nutritional interventions were managed by registered dietitians who assessed each patient’s and their family's dietary habits. Personalized nutrition plans were developed to address deficiencies and promote better health outcomes. These plans were reviewed and potentially adjusted at three months post-discharge during routine outpatient visits, ensuring sustained nutritional support.
3. *Social Support:* Social workers, in collaboration with nursing staff, facilitated access to community and healthcare resources. They assisted in arranging follow-up care and ensuring that patients received continuous support throughout their recovery period, including post-discharge.
4. *Oral Frailty Management:* Evaluations of swallowing function were conducted by otolaryngologists and speech therapists. These assessments aimed to prevent perioperative complications related to dysphagia and were integral during both the pre- and post-operative periods.
5. *Mental Health Support:* Certified psychiatric nurses provided mental health support, with additional resources such as psychiatric consultations made available as needed. This support was crucial for managing the psychological impact of cancer diagnosis and treatment, both before and after surgery.

The duration from the introduction of the CPAS program to gastrectomy depended on individual circumstances, including social factors and surgical wait times. The support services were adapted for home execution and were evaluated and tailored perioperatively. The formal CPAS program concluded at the patient's discharge, although post-discharge follow-up was part of the standard care protocol.

### Statistical analysis

The clinicopathological characteristics and short-term surgical outcomes of the two groups were compared using the chi-squared test for categorical variables and the Mann–Whitney U test for continuous variables. A *p*-value below 0.05 was deemed statistically significant. All statistical evaluations were conducted using JMP® PRO software (JMP version 16.1.0, SAS Institute, Cary, NC).

## Results

### Patient characteristics

**Fig. 1:**
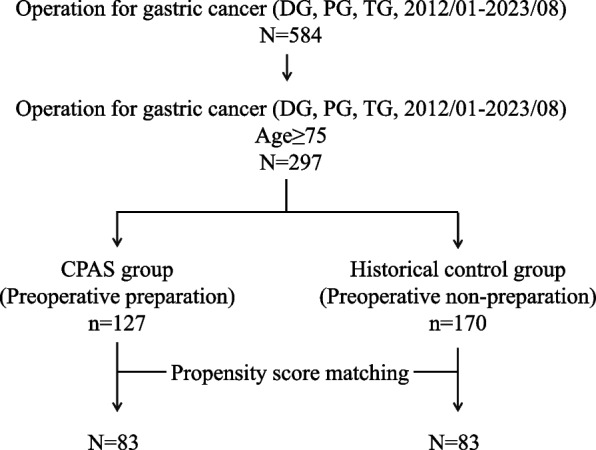
Study flow chart. Illustrates the patient selection from initial diagnosis to final analysis, detailing exclusions and the final cohort analyzed

After propensity score matching, 83 pairs of elderly gastric cancer patients were identified for analysis in both the CPAS and Historical control groups (Fig. 1). In the CPAS group (n = 83), the median age was 79, with a similar distribution of sex compared to the Historical control group (n = 83). Notably, the American Society of Anesthesiologists Physical Status (ASA-PS) score was significantly higher in the CPAS group (*P* < 0.001), indicating a potentially more compromised preoperative status. However, other baseline factors such as BMI, aCCI, clinical T status, clinical N status, and clinical Stage did not show significant differences between the groups, suggesting comparable overall health and cancer severity at baseline (Table 1).

**Table 1:** Comparative baseline characteristics of CPAS and control group

	After propensity score matching			Before propensity score matching		
	CPAS group (n = 83)	Control group (n = 83)	*p* value	CPAS group (n = 127)	Control group (n = 170)	*p* value
Age	79 (75–94)	79 (75–93)	0.51	80 (75–94)	80 (75–95)	0.83
Sex			0.87			0.62
Male	58 (69.9%)	57 (68.7%)		90 (70.9%)	116 (68.4%)	
Female	25 (30.1%)	26 (31.3%)		37 (29.1%)	54 (31.8%)	
ASA-PS			< 0.001			< 0.001
1	0 (0%)	6 (7.2%)		0 (0%)	7 (4.1%)	
2	50 (60.2%)	66 (79.5%)		77 (60.6%)	127 (74.7%)	
3	33 (39.8%)	11 (13.3%)		47 (37.0%)	35 (20.6%)	
4	0	0		3 (3.4%)	1 (0.6%)	
BMI (kg/m2)	21.9 (16.6–33.0)	22.1 (14.7–33.3)	0.78	22.23 (16.6–33.0)	22.0 (14.7–33.3)	0.054
aCCI	5 (4 – 10)	5 (4 – 11)	0.71	5 (4 – 10)	6 (4 – 11)	0.90
Clinical T status			0.76		56 (32.9%)	0.95
T1	37 (44.6%)	38 (45.8%)		38 (29.9%)		
T2	11 (13.3%)	15 (18.1%)		21 (16.5%)	27 (15.9%)	
T3	18 (21.7%)	14 (16.9%)		30 (23.62%)	40 (23.5%)	
T4	17 (20.5%)	16 (19.3%)		38 (29.92%)	47 (27.7%)	
Clinical N status			0.87			0.33
N +	26 (31.3%)	25 (30.1%)		55 (43.3%)	64 (37.7%)	
N-	57 (68.7%)	58 (69.9%)		72 (56.7%)	106 (62.4%)	
Clinical Stage			0.87			0.79
1	44 (53.0%)	49 (59.0%)		52 (40.9%)	74 (43.5%)	
2	16 (19.3%)	13 (15.7%)		25 (22.4%)	38 (22.4%)	
3	17 (20.5%)	16 (19.3%)		40 (28.2%)	48 (28.2%)	
4	6 (7.2%)	5 (6.0%)		10 (7.9%)	10 (5.9%)	

Values are presented as median (range) (*) or number (%). *P* = 0.05 was considered statistically significant. *ASA-PS* American Society of Anesthesiologists Physical Status, *BMI* Body mass index, *aCCI* Age-adjusted Charlson Comorbidity Index

### Spectrum of support and education in the CPAS group

**Fig. 2:**
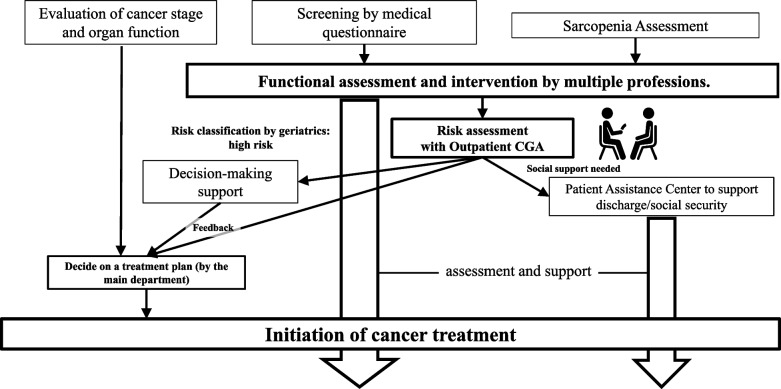
Flow chart of comprehensive preoperative assessment and support

In the CPAS group, sarcopenia was evaluated in 32 cases (38.6%), while 51 cases (61.4%) were non-sarcopenic (Fig. 2). Notably, 16 cases (19.3%) of the sarcopenic patients were identified as having severe sarcopenia. Of the overall sarcopenic patients, 33 patients (39.8%) received rehabilitation support, 25 (30.1%) nutritional support, 16 (19.3%) social support, 10 (12.0%) were assessed for oral frailty, and 6 (8.3%) received mental support (Table 2). These supports aimed at optimizing the patients' overall health and readiness for surgery, addressing the multifaceted needs of elderly patients facing major oncological surgery. Following the comprehensive preoperative assessment through the CPAS program, all patients assessed were deemed eligible for surgery, provided they could tolerate general anesthesia. There were no instances where CPAS assessments led to a decision against surgical intervention.

**Table 2:** Spectrum of Support and Education in the CPAS Group

	CPAS group (n = 83)
Rehabilitation support	33 (39.8%)
Nutritional support	25 (30.1%)
Social support	16 (19.3%)
Oral frail	10 (12.0%)
Mental support	6 (8.3%)

Values are presented as number (%)

### Short-term surgical outcomes

The short-term surgical outcomes showed significant differences between the groups (Table 3). The extent of lymph node resection was higher in the CPAS group (*P* = 0.005), indicating a more aggressive surgical approach compared to the Historical control group. However, there were no significant differences in the type of resection (TG vs. non-TG) or surgical approach (OS vs. MIS) between the groups. The operation time did not differ significantly (*P* = 0.13), but the median operative blood loss was significantly lower in the CPAS group (100 ml vs. 190 ml, *P* = 0.026), suggesting a potential benefit of the preoperative preparations.

**Table 3:** Details of Surgical Interventions and Immediate Postoperative Outcomes

	CPAS group (n = 83)	Control group (n = 83)	*p* value
Operative procedure			0.34
TG	15 (18.1%)	20 (24.1%)	
Non-TG (DG / PG)	68 (81.9%) (DG: 68/ PG: 0)	63 (75.9%) (DG: 56/ PG: 7)	
Approach			0.87
OS	34 (41.0%)	33 (40.0%)	
MIS	49 (59.0%)	50 (60.2%)	
Extent of node resection			0.005
D0/1	5 (6.0%)	15 (18.8%)	
D1 +	34 (41.0%)	41 (51.3%)	
D2	44 (53.0%)	24 (30.0%)	
Operation time (min)	285 (132–617)	307 (131–458)	0.13
Operative blood loss (ml)	100 (0–5931)	190 (0–1650)	0.026

Values are presented as median (range) (*) or number (%). *P* = 0.05 was considered statistically significant

*DG* Distal gastrectomy, *PG* Proximal gastrectomy, *TG* Total gastrectomy, *OS* Open surgery, *MIS* Minimal invasive surgery

Complications categorized as Clavien-Dindo > II were significantly lower in the CPAS group (19.3% vs. 33.7%, *P* = 0.034), indicating a reduction in serious postoperative complications (Table 4). Specifically, infection-related complications were significantly lower in the CPAS group, including respiratory complications (2.4% vs. 10.8%, *P* = 0.024) and superficial surgical site infections (1.2% vs. 9.6%, *P* = 0.011). These findings suggest that comprehensive preoperative support may contribute to a reduction in postoperative infectious complications, which are a significant concern in the elderly population. Notably, there were no postoperative mortalities in either group. The median postoperative hospital stay was significantly shorter in the CPAS group (10 days vs. 15 days, *P* < 0.001), highlighting the potential for enhanced recovery through targeted preoperative preparation.

**Table 4:** Postoperative hospital stay and complication rates

		CPAS group (n = 83)	Control group (n = 83)	*p* value
Postoperative hospital stays (days)		10 (7—101)	15 (4—141)	< 0.001
Overall surgical complications		17 (20.5%)	30 (36.1%)	0.024
Clavien-Dindo ≥ II		16 (19.3%)	28 (33.7%)	0.034
Clavien-Dindo ≥ III		6 (7.2%)	12 (14.5%)	0.13
Infectional complications		7 (8.4%)	22 (26.5%)	0.002
Respiratory disease		2 (2.4%)	9 (10.8%)	0.024
Surgical site infection		4 (4.8%)	13 (15.7%)	0.018
Details of SSI	Superficial incisional SSI	1 (1.2%)	8 (9.6%)	0.011
	Deep incisional SSI	4 (4.8%)	5 (6.0%)	0.73
Anastomotic leak		4 (4.8%)	3 (3.6%)	0.70
Pancreatic fistula		5 (6.0%)	2 (2.4%)	0.23
Ileus		5 (6.0%)	3 (3.6%)	0.47
Post operative bleeding		1 (1.1%)	2 (2.4%)	0.56
Delayed gastric emptying		2 (2.4%)	4 (4.8%)	0.40
Re-operation		1 (1.2%)	3 (3.6%)	0.30
Postoperative mortality (< 30 days)		0 (0%)	0 (0%)	-

Values are presented as numbers (%). *P* = 0.05 was considered statistically significant

SSI: surgical site infection

## Discussion

The recent global demographic shift towards an older population has led to an increased incidence of gastric cancer among the elderly. This demographic presents unique challenges due to the higher prevalence of comorbidities and the increased risk of postoperative complications [4–7]. Our study aimed to address these challenges by implementing CPAS strategy, including evaluations for frailty and sarcopenia. The results indicated that despite a higher ASA-PS score in the CPAS group, suggesting more complex cases, the group experienced less blood loss, fewer complications, particularly infectious and respiratory complications, and a shorter postoperative hospital stay. These findings underscore the potential of CPAS in improving short-term surgical outcomes for elderly gastric cancer patients. The CPAS group not only underwent more frequent D2 lymph node dissections but also exhibited significantly lower operative blood loss. This outcome underscores the impact of comprehensive preoperative assessments on surgical decision-making and execution. Enhanced surgical precision and the use of advanced intraoperative techniques, fostered by the detailed health profiles developed through CPAS, facilitated more extensive yet less invasive procedures. These findings highlight the integral role of meticulous preoperative planning in improving surgical safety and efficiency.

The role of comprehensive preoperative assessment in elderly gastric cancer patients is pivotal. Frailty and sarcopenia, prevalent in this demographic, are associated with increased postoperative morbidity and mortality. Studies have shown that preoperative identification and management of these conditions can significantly improve surgical outcomes [25, 26]. Our findings are consistent with recent research indicating that supports tailored to address frailty and sarcopenia, such as personalized exercise programs and nutritional supplementation, can enhance patient resilience and recovery [27, 28]. Furthermore, the comprehensive geriatric assessment has been recognized as a valuable tool for evaluating the physiological reserve and vulnerability of elderly patients, leading to more informed surgical decision-making and better alignment of patient and family expectations [29].

Our study underscores the efficacy of a multidisciplinary approach in managing elderly gastric cancer patients. The integration of medical, nutritional, psychological, and social support is crucial for addressing the complex needs of this population. Research has demonstrated that multidisciplinary care models, which include the collaboration of surgeons, geriatricians, nutritionists, and physiotherapists, lead to improved postoperative outcomes and patient satisfaction [30–32]. The CPAS program significantly contributed to improving patient awareness, particularly regarding their comprehensive health status. Patients became more aware of their overall conditions, including both cancer and non-cancer-related comorbidities. This enhanced awareness is essential for empowering patients, enabling them to actively participate in their own care and decision-making processes. Such improvements, while not quantitatively assessed in this study, are believed to contribute positively to patient outcomes in the long term.

Specifically, nutritional support has been shown to significantly reduce postoperative complications and enhance recovery, while psychological and social supports can help patients cope better with the stress of cancer and its treatment [29, 33]. The success of such models highlights the importance of a patient-centered approach that goes beyond the traditional focus on the surgical procedure itself, aiming to improve overall well-being and quality of life.

The reduction in postoperative complications, particularly infectious and respiratory complications, has significant implications for the long-term care of elderly gastric cancer patients. Studies have consistently shown that postoperative complications are associated with decreased long-term survival and increased long-term morbidity [34–39]. Our findings align with research suggesting that comprehensive preoperative supports can lead to a substantial reduction in these complications, thereby improving not only short-term outcomes but also long-term prognosis [40–42]. Moreover, the observed shorter hospital stays in the CPAS group align with studies indicating that enhanced recovery protocols, which include preoperative optimization, can expedite patient recovery and reduce healthcare costs [43, 44]. These benefits are particularly crucial for the elderly, for whom independence and quality of life are primary concerns.

Although our study offers valuable insights, it has several limitations. Firstly, being a single-center study, the findings may not be generalizable to all settings. Secondly, the retrospective nature of the study introduces potential biases. Thirdly, the sample size, though adequate for matching, is relatively small, which might limit the statistical power to detect differences in less common outcomes. Fourthly, the historical control group may not accurately represent current standard care due to changes in medical practice over time. Fifthly, the subjective nature of some assessments, such as frailty, might introduce variability. Sixthly, we did not account for long-term survival and quality of life, which are crucial for understanding the full impact of CPAS. Seventhly, Although the CPAS program led to notable improvements in patient awareness about their health conditions, these outcomes were not directly measured within the scope of this study. Future research should consider incorporating metrics that evaluate changes in patient awareness and understanding as part of the assessment of CPAS efficacy, providing a more comprehensive evaluation of its impact. Lastly, the economic implications of CPAS were not assessed, which is important for healthcare providers and policymakers. The retrospective design may have influenced the results, and a multi-center approach could provide more generalizable data.

## Conclusions

Our study demonstrates that comprehensive preoperative assessment and support significantly improve short-term surgical outcomes in elderly gastric cancer patients. The findings advocate for a multidisciplinary approach to preoperative preparation, emphasizing the importance of addressing frailty, sarcopenia, and other comorbidities. Future research should focus on larger, multicenter studies to confirm these results and explore the long-term impacts of CPAS on survival and quality of life. Additionally, studies assessing the cost-effectiveness of CPAS would provide valuable information for healthcare systems. Ultimately, the goal is to develop standardized protocols for the preoperative management of elderly gastric cancer patients, ensuring that they receive the best possible care tailored to their unique needs.

## Data Availability

No datasets were generated or analysed during the current study.

## Abbreviations

CPAS: Comprehensive preoperative assessment and support
DG: Distal gastrectomy
PG: Proximal gastrectomy
TG: Total gastrectomy
OS: Open surgery
MIS: Minimally invasive surgery
aCCI: Age-adjusted Charlson Comorbidity Index
BMI: Body mass index
CGA: Comprehensive geriatric assessments
